# Genome wide meta-analysis of cDNA datasets reveals new target gene signatures of colorectal cancer based on systems biology approach

**DOI:** 10.1186/s40709-020-00118-1

**Published:** 2020-06-08

**Authors:** Umair Ilyas, Shaiq uz Zaman, Reem Altaf, Humaira Nadeem, Syed Aun Muhammad

**Affiliations:** 1 Department of Pharmaceutics, Faculty of Pharmaceutical Sciences, Riphah International University, Islamabad, 44000 Pakistan; 2 Department of Pharmaceutical Chemistry, Faculty of Pharmaceutical Sciences, Riphah International University, Islamabad, 44000 Pakistan; 3 Institute of Molecular Biology and Biotechnology, Bahauddin Zakariya University, Multan, 66000 Pakistan

**Keywords:** Colorectal cancer, Microarrays, Functional genomics, PPI network, Biomarkers

## Abstract

**Background:**

Colorectal cancer is known to be the most common type of cancer worldwide with high disease-related mortality. It is the third most common cancer in men and women and is the second major cause of death globally due to cancer. It is a complicated and fatal disease comprising of a group of molecular heterogeneous disorders.

**Results:**

This study identifies the potential biomarkers of CRC through differentially expressed analysis, system biology, and proteomic analysis. Ten publicly available microarray datasets were analyzed and seven potential biomarkers were identified from the list of differentially expressed genes having a *p* value < 0.05. The expression profiling and the functional enrichment analysis revealed the role of these genes in cell communication, signal transduction, and immune response. The protein–protein interaction showed the functional association of the source genes (CTNNB1, NNMT, PTCH1, CALD1, CXCL14, CXCL8, and TNFAIP3) with the target proteins, such as AXIN, MAPK, IL6, STAT, APC, GSK3B, and SHH.

**Conclusion:**

The integrated pathway analysis indicated the role of these genes in important physiological responses, such as cell cycle regulation, WNT, hedgehog, MAPK, and calcium signaling pathways during colorectal cancer. These pathways are involved in cell proliferation, chemotaxis, cellular growth, differentiation, tissue patterning, and cytokine production. The study shows the regulatory role of these genes in colorectal cancer and the pathways that can be effected after the dysregulation of these genes.

## Background

After breast and lung cancer, colorectal cancer (CRC) has been diagnosed to be the third most common malignancy. It is the fourth leading cause of death with 1.4 million cases and almost 694,000 deaths. CRC is the third most common malignancy in males after prostate and lung cancer and second in females after breast cancer. The incidence rate of CRC has been rising in the developing countries due to westernization that is causing increased risk factors for CRC [1]. About 60% increase in the global burden of CRC, based on the demographic projections, is estimated to occur with 2.2 million new cases and 1.1 million deaths by 2030. More than 25% of patients with colorectal cancer are diagnosed with metastatic disease. Therefore, for improved sensitivity and specificity of detection of CRC new biomarkers have been developed [2]. Numerous risk factors are known to be associated with the progression of CRC with 95% of cases having adenocarcinomas. This includes enhanced alcohol intake, reduced physical exercise, a poor diet plan that is rich in fats and poor in fibers, personal or familial history of polyps, age greater than 50, and inflammatory bowel disease [3].

Following the development of colorectal carcinoma, the subsequent genetic and epigenetic alterations in specific oncogenes and/or tumor suppressor genes of gastrointestinal epithelial cells causes it to undergo cell proliferation and self-renewal, triggering the normal epithelium to be hyperproliferative mucosa. This results in a benign adenoma that eventually grows into carcinoma and in about 10 years becomes metastatic [4].

The normal epithelial cells of the gastrointestinal tract are arranged along a crypt-villus axis. The undifferentiated pool of colon stem cell and progenitor cells having the ability of self-renewal and pluripotency are found at the bottom of the crypt. These cells while moving along the axis undergo differentiation in all epithelial colon lineages. Whilst these cells arrive at the top of the axis which usually takes 14 days they result in apoptosis. Several proteins are known to be involved in the regulation of this process such as BMP, Wnt, and TGF-β [4]. The onset of CRC has shown the involvement of various altered molecular signaling pathways that may result in the resistance to anti-tumor agents. These pathways include the Wnt/APC/β-catenin, transforming growth factor-β (TGF)-β/Smad, phosphoinositide 3-kinase (PI3K)/AKT/glycogen synthase kinase-3B and NF-κβ.

The diagnosis of CRC plays a pivotal role in the early prediction of CRC. If detected early it can be treated with surgery alone, however, in metastatic disease along with surgery chemotherapy is included. Presently, the prediction of CRC is based on the classification of the American Joint committee on Cancer (AJCC), TNM staging. But because each stage is a heterogeneous group of disease it is difficult to relate the TNM staging with prognosis. A more rapid and cheaper form of molecular characterization of cancer has become possible with the advancement of NGS technology. Most of the genetic biomarkers have gained a clinical value as a prognostic or therapeutic marker such as the MSI and the EGF signaling pathway [4].

To get a clear picture of the carcinogenesis, tumor growth and metastasis of colorectal cancer, the microarray analysis has been proved useful to gather information on thousands of genes at a time. The genomic alterations occurring in colorectal cancer can be identified by microarray analysis which can help in the diagnosis, characterization, and treatment of colorectal cancer [5]. However, certain challenges are still faced in the application of microarray assays according to some studies. One approach to overcome such challenges is to utilize the online Gene Expression Omnibus (GEO) database. This database can assist in increasing the size of the sample, statistical power and sample heterogeneity [6–8].

The aim of this study is to screen out significant CRC associated genes that can act as candidate biomarkers to detect early cancer and to elucidate the pathogenesis of CRC. The differential expression analysis of ten microarray datasets was performed to identify the candidate genes based on significant scoring function. The expression profiling of these genes was also performed to determine the expression patterns of these potential markers in several tissues. Cluster analysis and Functional enrichment analysis was employed to confirm the function and association of shortlisted genes in causing CRC. The protein–protein interaction and pathway analysis confirmed the association of candidate genes with colorectal cancer and the regulation of Wnt, NF-κβ, and MAPK. The search for new predictive, diagnostic and prognostic biomarkers in colorectal cancer is of great importance and has become the goal of biomedical research on CRC. The study will aid in the biomarker discovery by getting valuable insights through studying these molecular networks that can be used in public datasets for better outcomes in other diseases as well.

## Methods

### Accession of gene expression data

The aim of this study was to identify potential targets for colorectal cancer. The gene expression datasets of colorectal cancer were accessed from the Gene Expression Omnibus database under two screening conditions (organism: *Homo sapiens*, experiment type: expression profiling by an array). Each dataset comprises of GEO accession number, platform, sample type, number of samples and gene expression data. The array platform used was Affymetrix GeneChip Human Genome U133 Plus 2.0 Array (CDF: Hs133P_Hs_ENST, version 10) (Affymetrix, Inc., Santa Clara, CA, 95051, USA, Technology: in situ oligonucleotides). In order to detect the gene expression, the array platform and the annotation information (hgu133plus2) of probes were used. Computational analysis was performed using R and BioConductor packages containing AffyQCReport, Affy, Annotate, AnnotationDbi, Limma, Biobase, AffyRNADegradation, hgu133plus2cdf, and hgu133a2cdf.

### Preprocessing and differential expression analysis of microarray datasets

The phenodata files were prepared for each dataset in a recognizable format [9]. The normalization of the data was done using Bioconductor “ArrayQuality Metrics” package on R version 3.1.3 to a median expression level of each gene [10–12]. This was done to compare the microarray data sets. The background correction for perfect matches (PM) and mismatches (MM) was performed by the Robust Multi-array Analysis (RMA) in order to remove local noise and artifacts [9]. Perfect matches (PM) and mismatches (MM) was calculated using the following equation.

$$ PM_{ijk} \, = \,BG_{ijk} \, + \,S_{ijk}. $$

where, PM is a perfect match, Background (BG) caused by optical noise and non-specific binding (S);* ijk* is the signal for probe* j* of probe set* k* on array* i*.

$$ BG\left( {PM_{ijk} } \right)\, = \,E\left[ {S_{ijk}\mid \,PM_{ijk} } \right]\, > {0}. $$

$$ S_{ijk} \,\sim \,Exp\left( {\lambda_{ijk} } \right)\,BG_{ijk} \,\sim \,N\left( {\beta_{i} ,\,\sigma^{2} } \right). $$

The background (BG) and the signal expression (E) forms the PM-data. The dataset normalized to median level expression was analyzed by the “Array Quality Metrics” package of Bioconductor software [10–12]. Expression value having a *p*-value < 0.15 was considered marginal log transformation. For each dataset, the gene–gene covariance matrix was calculated across all array (54675 Affyids) using the following formula.

$$ X_{norm} \, = \,F2^{ - 1} \,\left( {F1\left( x \right)} \right). $$

where *F*1 and *F*2 are distribution functions of the actual and reference chips, respectively.

To attain the summary of intensities the RMA-algorithm was used to calculate the averages between probes in a probe set.

The degradation analysis to measure the quality of RNA in these datasets, AffyRNADegradation package of Bioconductor was used (Affymetrix, 1999, 2001). The pairwise comparison was done to identify the DEGs in each dataset and for multiple testing correction Benjamini–Hochberg method was employed. The differentially expressed genes were shortlisted along with measurement of quality weights. The shortlisted genes were ranked according to their *p* values and the resulting scores. Significant cut off values was set to calculate the moderated statistics with *p*-value ≤ 0.05, FDR < 0.05 (false discovery rate), absolute log fold change (logFC) > 1 and FDR < 0.05 (false discovery rate) [13].

### Curation of CRC-related genes

The shortlisted DEGs were further screened for colorectal associated genes using diverse data source including PubMed, MeSH, OMIM, and PMC database to filter significant disease specific genes. The cluster analysis [14] was performed based on expression values in each dataset of CRC-related differential expressed genes in order to identify the variations in gene expression levels between treated and untreated replicates using CIMminer tool [15].

### Functional enrichment analysis

In order to understand the biological functions of these CRC related genes the Gene ontology, functional annotation and pathway enrichment analysis [16, 17] was performed. The web-based tools used for this purpose were DAVID (Database for Annotation Visualization and Integrated Discovery) [18] and FunRich Annotation tools [19].

### Protein–protein interaction network

The topology and functional protein interactions are useful to analyze the biological and pathological conditions of a specific disease. The protein–protein interaction helps in identifying these features and the functional relationship of such proteins can be interpreted through genomic associations [17, 20]. The PPI network shows the interaction of each protein with a number of other genes with biological or molecular functions having different activity in the pathological state as compared to normal [21]. Proteins that interacted with each other during colorectal cancer were evaluated from STRING (Search Tool for the Retrieval of Interacting Genes/Proteins) [21] and HAPPI databases (Human Annotated and Predicted Protein Interaction) databases [22] with a confidence score of 0.999. Cytoscape software (version 3.2.1) was used to visualize the molecular and network interaction to identify the role of seeder and target genes in CRC [23]. The network showed the role of each target gene signatures that interacted with CRC associated source genes in colorectal cancer. The role was determined by mapping the target gene with seeder genes using, OMIM, MeSH, and PMC databases. The gene mapping determines the potential colorectal related gene signatures to be functionally related whose dysregulation causes a disease phenotype. The total number of target genes interacted with each source protein was calculated. A molecular sub-network of those genes that were associated with pathways of interest causing colorectal cancer was constructed. The topological network properties were calculated using Network Analyzer in Cytoscape [24].

### Integrated pathway modeling

The integrated and metabolic networks of CRC-related source genes were analyzed and the Co-relation between test genes was observed. To recognize the underlying pathways involved in the progression of CRC the pathway analysis was performed which could help in identifying the biomarkers of this disease. The curation and mapping of candidate biomarkers were done using KEGG (Kyoto Encyclopedia of Genes and Genomes) [25], Reactome and Wiki pathways. PathVisio3tool was used to reconstruct the cellular and signaling pathways of potential biomarkers [26] and the potential mechanism of each marker in the pathway was studied based on the pieces of evidence available in literature and databases. For cross-verifications, we used TCGA and the Human Protein Atlas databases and analyzed the expression level of ranked DEGs in colorectal cancer.

## Results

### Microarray analysis and normalization

**Fig. 1:**
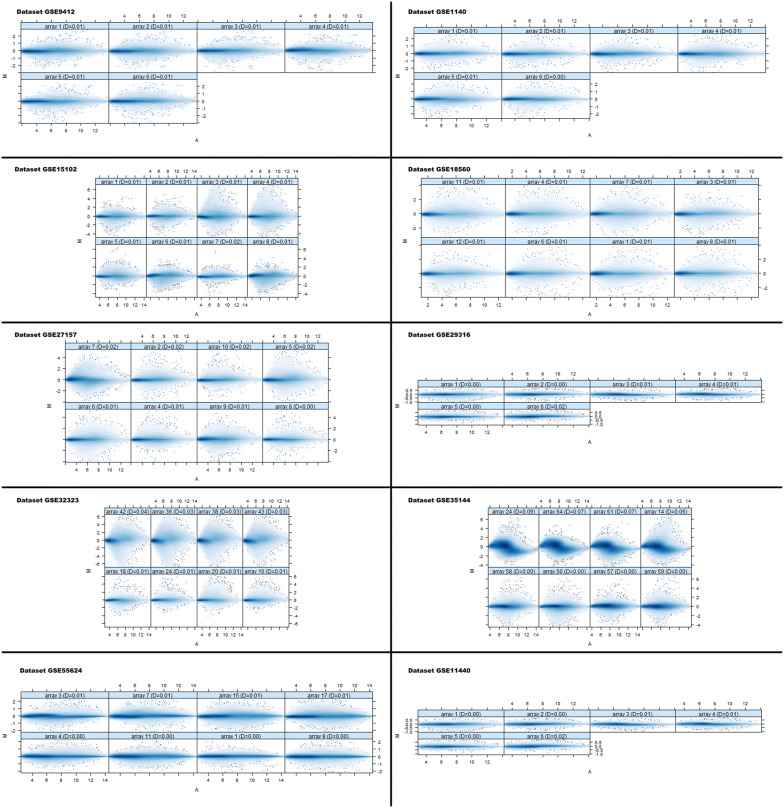
MA plots of an individual quality array after normalization. M and A is specified as $\text{M}\,\text{ = }\,\text{log}_{2} \,\left( {\text{I}_{1} } \right)\, - \,\text{log}_{2} \,\left( {\text{I}_{2} } \right),\,\text{A}\,\text{ = }\,\text{1/2}\,\,\left( {\text{log}_{2} \,\left( {\text{I}_{1} } \right)\, - \,\text{log}_{2} \,\left( {\text{I}_{2} } \right)} \right)$, where I_1_ is the intensity of the array studied, and I_2_ is the intensity of a “pseudo”-array that consists of the median across arrays”. Normally, the mass of distribution in the MA plot is expected to be concentrated along the M = 0 axis with no trend in M as a function of A

**Fig. 2:**
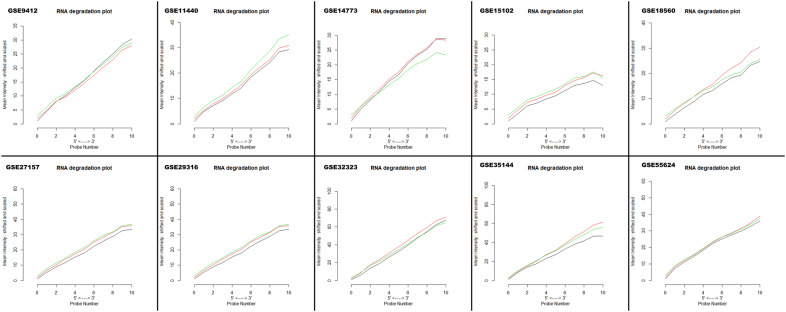
RNA degradation plot of each dataset produced by plot AffyRNAdeg showing 5′ to 3′ trend to evaluate the severity of RNA degradation and significance level

Ten datasets were downloaded from online GEO database with .CEL format related to CRC. The size of an array of AffyBatch object comprised of 1164 × 1164 and 732 × 732 features with related Affyids (Table 1). In order to avoid the systematic variation, the quantile normalization was used for background correction and normalization. The probe level data obtained after normalization shows the quality of the individual array of each dataset in the MA plots (Fig. 1). The computation of gene–gene covariance matrix across all arrays was performed by ignoring the missing values in each dataset and to assure they were on a similar scale to log–transform the arrays. In each probe sets, the probes were arranged by location relative to the 5′ end of the targeted RNA molecule. The severity of RNA-degradation and significance level was presented by function plotAffyRNAdeg (Fig. 2) and a single summary statistic for each array in the batch was produced by the function summary of AffyRNAdeg (Additional file 1: Table S1). The list of databases, tools, and software used in this study are available in (Additional file 1: Table S2).

**Table 1:** List of cDNA datasets

Accession No.	Total samples	Tissues	Species	Conditions/type	Platform	Size of arrays	AffyIDs	References
GSE 9412	6	Colorectal cancer	*Homo sapiens*	Sensitive vs resistant	GPL571 [HG-U133A_2] Affymetrix Human Genome U133A 2.0 Array	732x732 features	22277	Selga et al. [34]
GSE 11440	6	Colon	*Homo sapiens*	Sensitive vs resistant	GPL570 [HG-U133_Plus_2] U133 Plus 2.0 Array	1164x1164 features	54675	Mencia et al. [35]
GSE14773	8	Colon	*Homo sapiens*	Control vs treated	GPL570 [HG-U133_Plus_2] U133 Plus 2.0 Array	1164x1164 features	54675	Hwang et al. [36]
GSE 15102	8	Colon	*Homo sapiens*	Control vs treated	GPL571 [HG-U133A_2] Affymetrix Human Genome U133A 2.0 Array	732x732 features	22277	Sagiv et al. [37]
GSE 18560	12	Colorectal	*Homo sapiens*	Control vs treated	GPL570 [HG-U133_Plus_2] Affymetrix Human Genome U133 Plus 2.0 Array	1164x1164 features	54675	Selga et al. [34]
GSE 27157	10	Colorectal cancer	*Homo sapiens*	p53 wild vs. tumor samples	GPL570 [HG-U133_Plus_2] Affymetrix Human Genome U133 Plus 2.0 Array	1164x1164 features	54675	Katkoori et al. [38]
GSE 29316	6	Colon fibroblast	*Homo sapiens*	Control vs treated	GPL570 [HG-U133_Plus_2] Affymetrix Human Genome U133 Plus 2.0 Array	1164x1164 features	54675	Chen et al. [39]
GSE 32323	44	Colorectal cancer	*Homo sapiens*	Control vs Treated	GPL570 [HG-U133_Plus_2] Affymetrix Human Genome U133 Plus 2.0 Array	1164x1164 features	54675	Khamas et al. [40]
GSE 35144	64	Colorect cancer	*Homo sapiens*	Case vs control	GPL570 [HG-U133_Plus_2] Affymetrix Human Genome U133 Plus 2.0 Array	1164x1164 features	54675	Uronis et al. [41]
GSE 55624	18	Colorectal cancer	*Homo sapiens*	Control vs treated	GPL570 [HG-U133_Plus_2] Affymetrix Human Genome U133 Plus 2.0 Array	1164x1164 features	54675	Schoumacher et al. [42]

### Identification and screening of differentially expressed genes

About 50 DEGs in each microarray dataset were identified by pairwise comparison between biologically comparable groups. From these 50 DEGs, the top 20 genes in each dataset were ranked and selected based on FDR (< 0.05), *p*-value (≤ 0.05) and logFC (> 1) parameters. And from these 20 genes, seven common genes of each dataset were identified as the potential biomarker candidate (Additional file 1: Table S3).

### Data mining and cluster analysis

The seven significant colorectal cancer associated genes shortlisted from the differentially expressed genes were CALD1, CTNNB1, CXCL14, PTCH1, CXCL8, TNFAIP3, and NNMT after mapping with PubMed, OMIM, MeSH, and PMC databases. The role of sorted genes in colorectal cancer was curated and counted (Table 2).

**Table 2:** The differentially expressed CRC-associated genes curated from PubMed

S.NO.	Probe ID	Gene ID	Uniprot_ID	PubMed Count	Protein name	Reference links
1.	202237_at	NNMT	NNMT_HUMAN	4	NNMT *Homo sapiens* nicotinamide N-methyltransferase	https://www.ncbi.nlm.nih.gov/pubmed/?term=%22NNMT%22+AND+%22COLORECTAL+CANCER%22
2.	212077_at	CALD1	CALD1_HUMAN	5	Caldesmon 1	https://www.ncbi.nlm.nih.gov/pubmed/?term=%22+CALD1%22+AND+%22COLORECTAL+CANCER%22
3.	201533_at	CTNNB1	CTNB1_HUMAN	506	CTNNB1 Homo sapiens catenin beta 1	https://www.ncbi.nlm.nih.gov/pubmed/?term=%22+CTNNB1%22+AND+%22COLORECTAL+CANCER%22
4.	222484_s_at	CXCL14	CXL14_HUMAN	3	C-X-C motif chemokine ligand 14	https://www.ncbi.nlm.nih.gov/pubmed/?term=%22+CXCL14+%22+AND+%22COLORECTAL+CANCER%22
5.	209815_at	PTCH1	PTC1_HUMAN	15	Patched 1	https://www.ncbi.nlm.nih.gov/pubmed/?term=%22+PTCH1+%22+AND+%22COLORECTAL+CANCER%22
6.	202644_s_at	TNFAIP3	TNAP3_HUMAN	26	TNF alpha induced protein 3	https://www.ncbi.nlm.nih.gov/pubmed/?term=%22+TNFAIP3%22+AND+%22COLORECTAL+CANCER%22
7.	202859_x_at	CXCL8		5	C-X-C motif chemokine ligand 8	https://www.ncbi.nlm.nih.gov/pubmed/?term=%22+CXCL8+%22+AND+%22COLORECTAL+CANCER%22

**Fig. 3:**
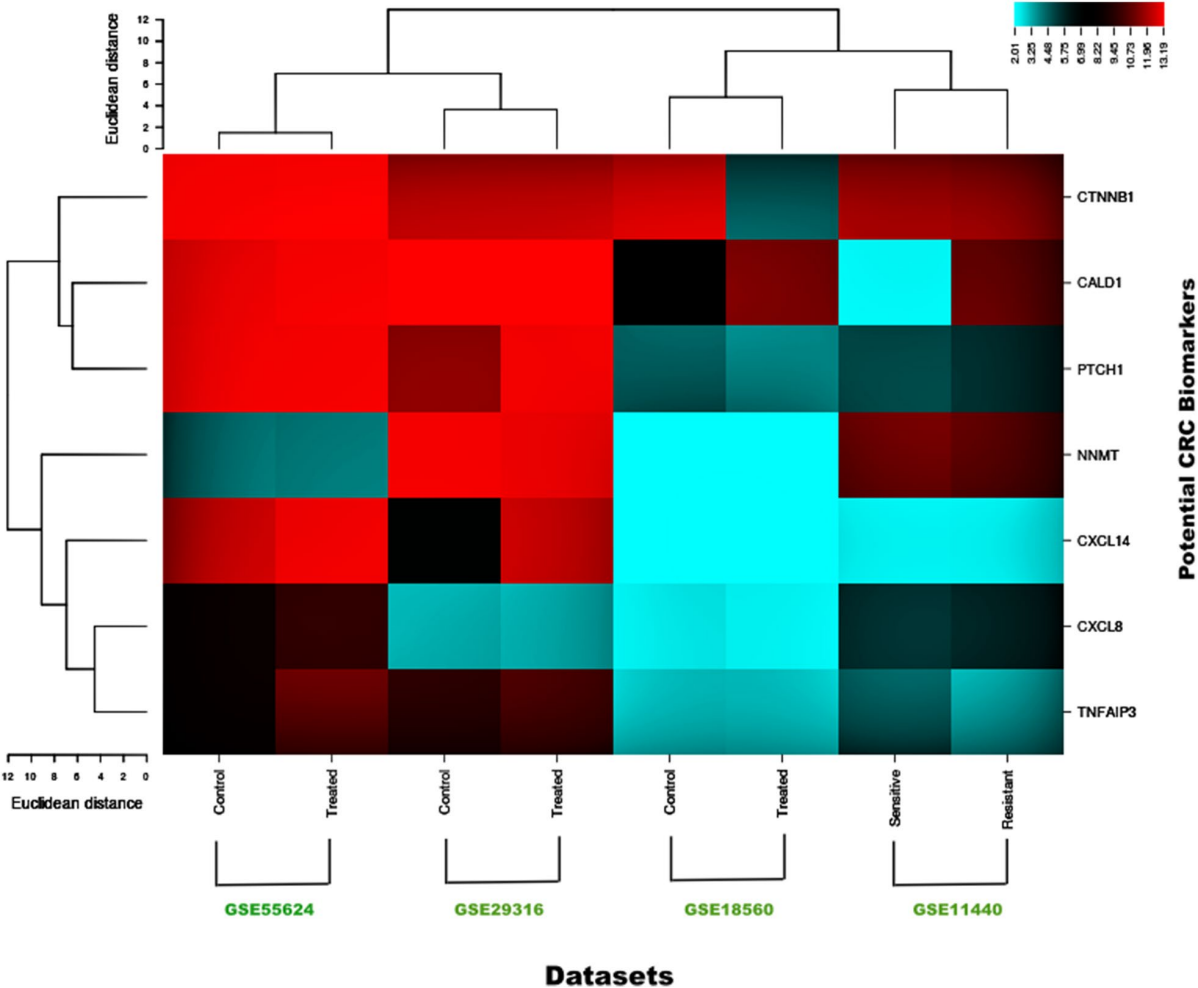
Cluster analysis of 7 colorectal related DEGs. Blue corresponds to a small distance and Red to a large distance. Lines indicate the boundaries of the clusters in the level of the tree

The genetic expression of colorectal cancer cell samples showed a clear difference between the treated and untreated groups (Fig. 3).

### Gene enrichment analysis

**Fig. 4:**
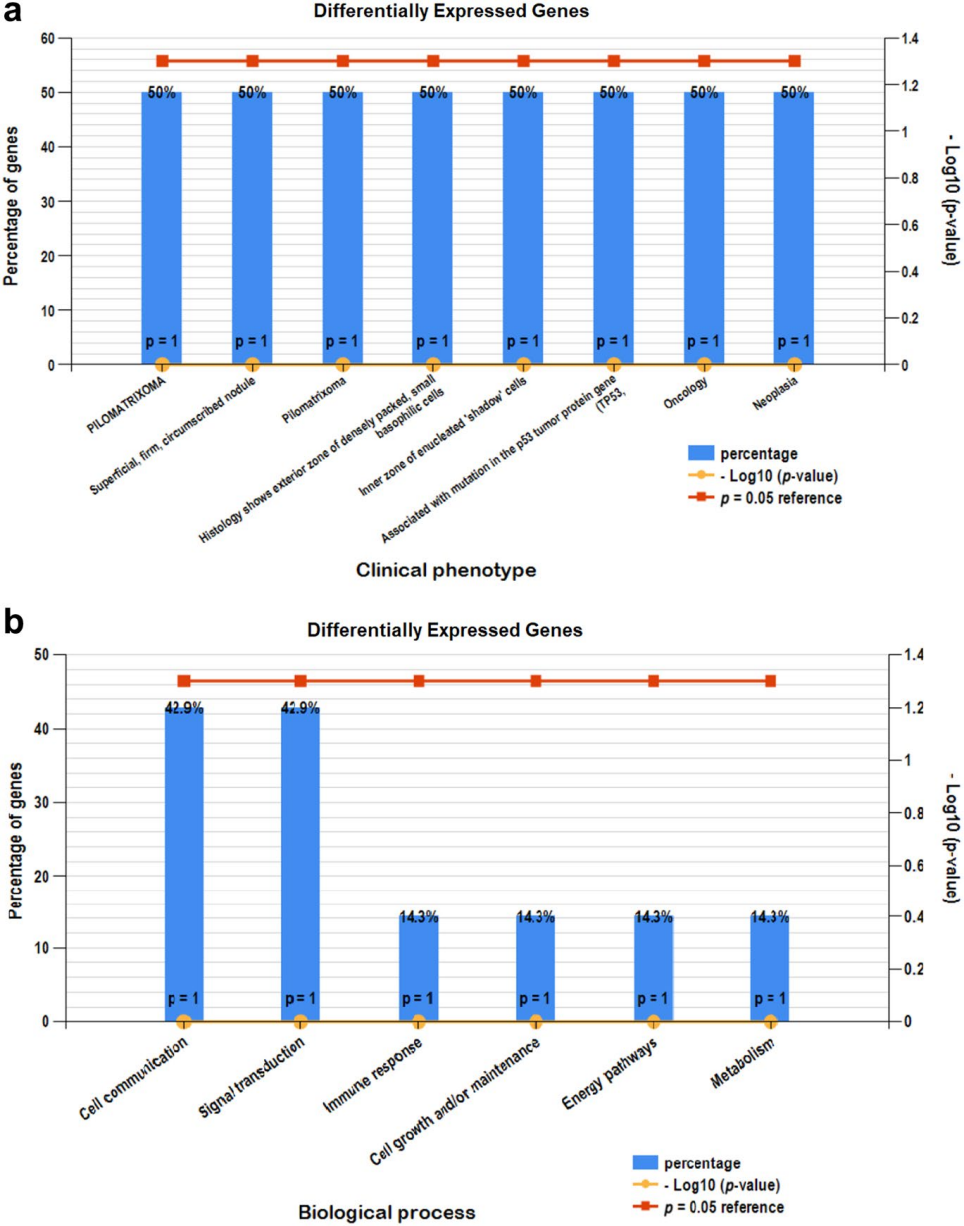
**a** Clinical phenotypes analysis of CRC related DEGs. **b** Biological pathway analysis using FunRich tool

Significant enrichment was obvious in 5 downregulated and 2 upregulated genes. The clinical phenotypes associated with the dysregulation of these genes are pilomatrixoma, congenital lung cyst and ovarian fibromata (Fig. 4a). The biological processes are related to cell communication, signal transduction, immune response, energy, metabolism and cell growth and maintenance (Fig. 4b).

### Gene network analysis

**Fig. 5:**
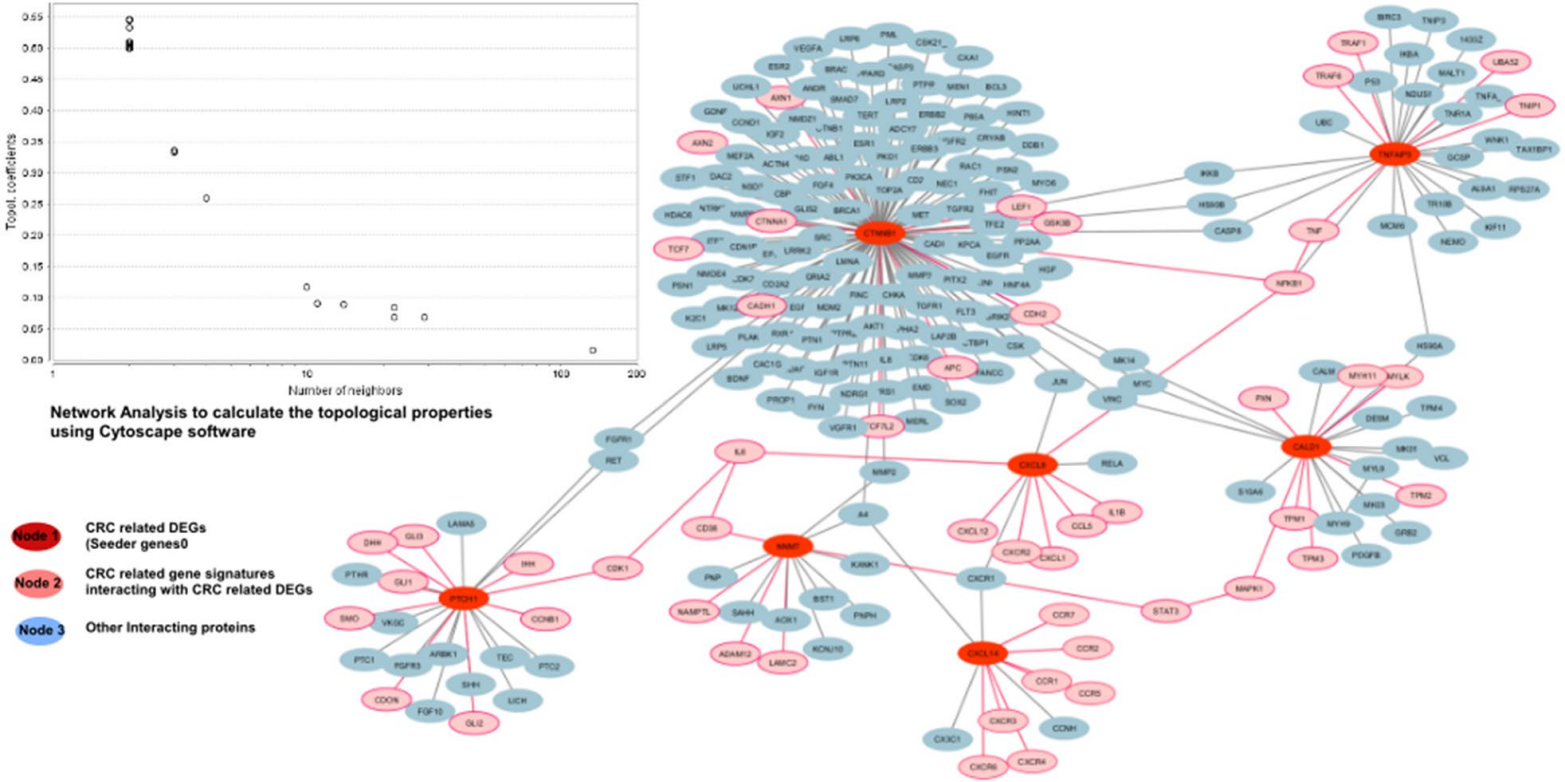
A genetic network of a total number of gene signatures associated with CRC differentially expressed seeder genes. Red nodes represent CRC seeder genes, blue nodes showing signature genes associated with seeder genes having no role in CRC while pink nodes represent gene signatures associated with seeder genes having a role in CRC

In PPI, a total of 233 nodes and 134 edges were retrieved from STRING [21] and HAPPI database [22] with a confidence score of 0.99. The database showed the interaction of CRC-associated genes with potential other genes that were contributing to a disease phenotype. The network was categorized into three neighborhoods: light pink and red nodes indicate the CRC-associated potential biomarkers while the remaining blue nodes represent the other target proteins. The potential biomarkers were found to functionally interact with other biologically essential target proteins. Some of them are, APC, IL6, MAPK1, NFkb1 and SHH (Fig. 5). The source protein CTNNB1 was shown to be interacting with APC and NNMT showed interaction with CDK38 and STAT3. Similarly, CALD1 is associated with MAPK1 while, PTCH1 shows interaction with a family of hedgehog proteins SHH, IHH and DHH with clinical phenotypes. The network analyzer was used to classify and improve the network performance and to interpret the topological properties of the network. The disease gene mapping of target genes using CTD showed that more than 50 genes have a functional relation with the source/seeder genes in CRC (Fig. 5).

### Pathway modeling

**Fig. 6:**
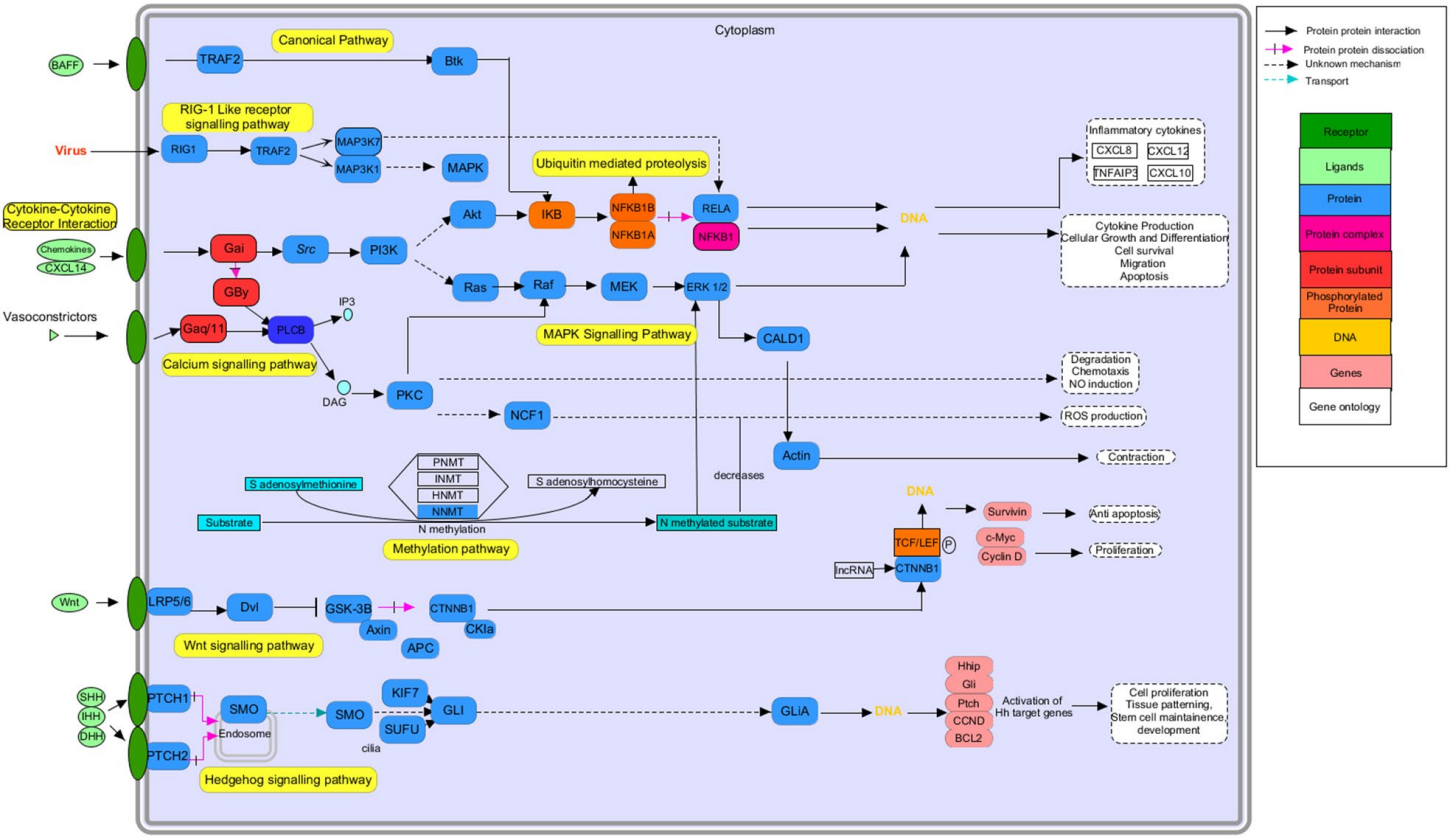
Pathway modeling. Integrated genome to phenome scale signaling pathways involved in CRC. KEGG pathway was used to map the gene signatures for signaling and metabolic reconstruction

**Fig. 7:**
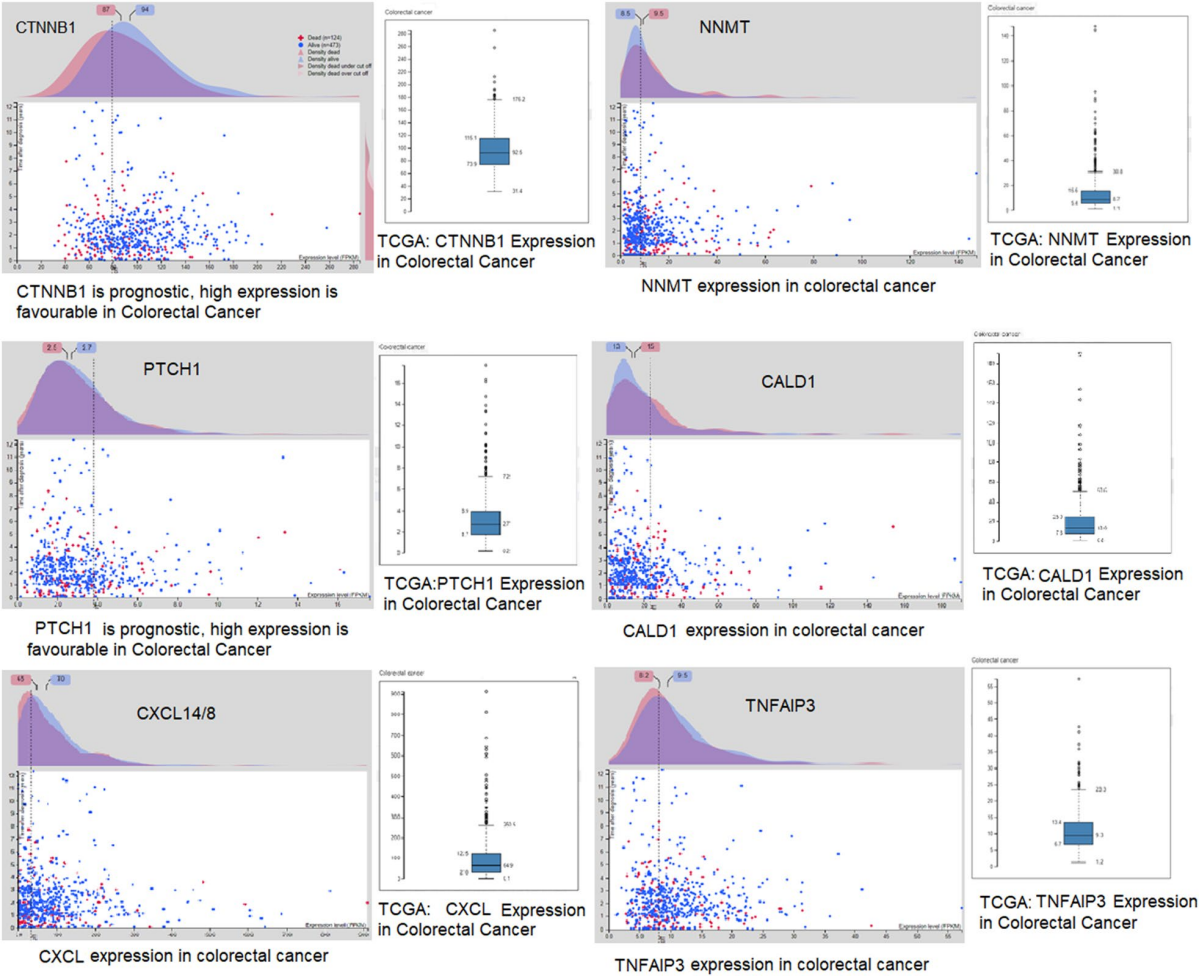
Interactive scatter plot indicates the expression-level of differentially expressed genes in colorectal cancer. These plots showed the consequence of RNA and protein levels on clinical survival

The source genes identified were further studied to evaluate the molecular mechanism of these genes in CRC. The network generated after reconstruction showed that several pathways were involved in the pathogenesis of colorectal cancer. Along with the Wnt pathway and the canonical pathway other pathways, the MAPK pathway, Calcium signaling pathway, metabolic pathway and RIG-like 1 receptor pathway have also shown a connection with colorectal cancer (Fig. 6). The gene ontology of these pathways is associated with cell proliferation, chemotaxis, stem cell maintenance, and apoptosis. Therefore, the progression of CRC is related to the overexpression of genes that leads to cell proliferation and anti-apoptosis and downregulation of genes that inhibit the proliferation and cellular differentiation of cells. The association of differentially expressed genes with colorectal cancer were cross-referenced by TCGA and the Human Protein Atlas. The median expression level of interactive gene signatures and source DEGs is significant in cases as compared to control. The interactive survival scatter plot (Fig. 7) indicates the expression of these DEGs is favorable in colorectal cancer. The pathways and pathological analysis showed that the proteins level is linked with cancer. These interactive survival scatter plots indicated the consequence of RNA and protein levels on clinical survival. The results showed that individual tumor gene expression patterns differed greatly and could surpass the variability between different types of cancer. Lower patient survival was usually associated with over-expression of genes in mitosis and cell growth and down-regulation of genes involved in cellular differentiation. This data enables the generation of metabolic models in a customized genome scale for cancer patients to recognize important genes of tumor growth.

## Discussion

In this study, the DEG for colorectal cancer were identified and their GO studies were performed to analyze their functions. The gene network was established and the pathways through which these DEGs were deregulated were also identified. The study provides a new platform for the determination of pathogenesis of colorectal cancer.

The differential analysis revealed 7 differentially expressed signature genes out of 50 DEGs on the basis of physicochemical and functional analysis (*p* < 0.05) that showed involvement in the progression of colorectal cancer. These signature genes are CALD1, CTNNB1, CXCL14, PTCH1, CXCL8, TNFAIP3, and NNMT. The functional role of these genes in colorectal cancer and their dysregulation has been extensively studied [27–30]. Out of seven genes, two genes were upregulated while the rest showed downregulation. The GO study revealed that following functional categories were enriched among dysregulated genes; response to molecule of bacterial origin, response to a drug, cellular response to lipopolysaccharide, chemotaxis, movement of a cell or subcellular component and branching involved in ureteric bud morphogenesis. These genes showed a link to important biological pathways such as, methylation, beta-catenin signaling cascade and wnt canonical pathway. The dysregulation of these genes can cause Pilomatrixoma, congenital lung cyst, and other clinical phenotypes.

The network study showed the functional association of possible biomarker candidates with other interacting protein targets such as APC, AXIN, MAPK, TRAF, GLI, and SHH. The mutation of APC genes has been shown to be present in 90% of the patients with human CRC that interacts with CTNNB1 in the Wnt pathway. More than 80 target genes showed a connection with the source genes in causing colorectal cancer. The mutation of Kras genes also plays a pivotal role in the progression of colorectal cancer during the early adenoma stage. The cross talk between the wnt/β-catenin and RAS-ERK pathway exist and the interaction between the two pathways during the various stages of colorectal cancer the combined mutations of which lead to malignant transformation of CRC [31]. The interaction between the NNMT and the MAP/ERK pathway has also been under investigation reporting the cell survival, apoptosis and cell cycle progression of cancer tissues. The overexpression of NNMT has indicated the acceleration of cell proliferation by regulating the energy metabolism in CRC tissues and by its involvement with the P13K/Akt and MAP/ERK pathways [27]. The GLi and SHH are part of the hedgehog signaling pathway that plays an essential role in the differentiation, growth, tissue patterning and cell maintenance of various cancers. However, its role in the CRC is still controversial [32]. PTCH1 is a tumor suppressor gene and its downregulation causes the activation of GLi transcription factors that activate the hedgehog target genes. CXCL14 is another potential tumor inhibiting gene in colorectal carcinoma and downregulation of this gene may result in a more aggressive phenotype of colorectal cancer [30]. Another prognostic biomarker of colorectal cancer is the TNFAIP3 which may also act as the tumor suppressor gene [33].

The network analysis revealed that these biomarkers play an essential role in colorectal cancer and that the dysregulation of these genes may lead to the progression of cancer. Targeting these pathways and the genes involved in the signaling of these pathways may help in easing the therapeutic profiling of colorectal cancer. The CTNNB1 and NNMT are known targets of CRC and both genes are known to interact with the MAPK pathways in causing CRC. The integrated network-based analysis helped in identifying the interaction of these potential biomarkers with other target genes through different integrated pathways.

## Conclusion

Advancement in the clinical therapy of diseases is still a requisite and to improve the diagnostic measures validated biomarkers can aid in the diagnosis. The microarray analysis has helped in several ways to identify the novel targets for targeted based therapies. This study reveals the essential biomarkers involved in CRC through a system biology approach. Seven essential biomarkers of CRC having functional relation with other important target proteins such as APC, MAPK and GLi and have found a significant association with CRC. The study might help in rapid risk assessment of colorectal cancer by providing the new insights in clinical practice utilizing the microarray gene expression analysis.

## Supplementary information

**Additional file 1: Table S1.** The function summaryAffyRNAdeg of Bioconductor package produced a single summary-statistic for each array in the batch dataset.

**Additional file 2: Table S2.** List of databases, software, and tools used in this study.

**Additional file 3: Table S3.** Preliminary investigation of common and related differentially expressed genes of each microarray dataset.

## Data Availability

The data has been presented with the article.
