# Correction to: Genome wide meta-analysis of cDNA datasets reveals new target gene signatures of colorectal cancer based on systems biology approach

**DOI:** 10.1186/s40709-020-00121-6

**Published:** 2020-07-06

**Authors:** Umair Ilyas, Shahiq uz Zaman, Reem Altaf, Humaira Nadeem, Syed Aun Muhammad

**Affiliations:** 1 Department of Pharmaceutics, Faculty of Pharmaceutical Sciences, Riphah International University, Islamabad, 44000 Pakistan; 2 Department of Pharmaceutical Chemistry, Faculty of Pharmaceutical Sciences, Riphah International University, Islamabad, 44000 Pakistan; 3 Institute of Molecular Biology and Biotechnology, Bahauddin Zakariya University, Multan, 66000 Pakistan

## Correction to: J Biol Res-Thessaloniki (2020) 27:8 10.1186/s40709-020-00118-1

Following publication of the original article [1], the authors identified an error in the author name of Shahiq uz Zaman.

The incorrect author name is: Shaiq uz Zaman.

The correct author name is: Shahiq uz Zaman.

The author group has been updated above and the original article [1] has been corrected.
